# Neuropathogenesis of Zika Virus in a Highly Susceptible Immunocompetent Mouse Model after Antibody Blockade of Type I Interferon

**DOI:** 10.1371/journal.pntd.0005296

**Published:** 2017-01-09

**Authors:** Darci R. Smith, Bradley Hollidge, Sharon Daye, Xiankun Zeng, Candace Blancett, Kyle Kuszpit, Thomas Bocan, Jeff W. Koehler, Susan Coyne, Tim Minogue, Tara Kenny, Xiaoli Chi, Soojin Yim, Lynn Miller, Connie Schmaljohn, Sina Bavari, Joseph W. Golden

**Affiliations:** 1 Virology Division, United States Army Medical Research Institute of Infectious Diseases, Fort Detrick, Maryland, United States of America; 2 Pathology Division, United States Army Medical Research Institute of Infectious Diseases, Fort Detrick, Maryland, United States of America; 3 Molecular and Translational Sciences Division, United States Army Medical Research Institute of Infectious Diseases, Fort Detrick, Maryland, United States of America; 4 Diagnostics Systems Division, United States Army Medical Research Institute of Infectious Diseases, Fort Detrick, Maryland, United States of America; 5 Veterinary Medicine Division, United States Army Medical Research Institute of Infectious Diseases, Fort Detrick, Maryland, United States of America; 6 Headquarters Division, United States Army Medical Research Institute of Infectious Diseases, Fort Detrick, Maryland, United States of America; University of California, Berkeley, UNITED STATES

## Abstract

Animal models are needed to better understand the pathogenic mechanisms of Zika virus (ZIKV) and to evaluate candidate medical countermeasures. Adult mice infected with ZIKV develop a transient viremia, but do not demonstrate signs of morbidity or mortality. Mice deficient in type I or a combination of type I and type II interferon (IFN) responses are highly susceptible to ZIKV infection; however, the absence of a competent immune system limits their usefulness for studying medical countermeasures. Here we employ a murine model for ZIKV using wild-type C57BL/6 mice treated with an antibody to disrupt type I IFN signaling to study ZIKV pathogenesis. We observed 40% mortality in antibody treated mice exposed to ZIKV subcutaneously whereas mice exposed by intraperitoneal inoculation were highly susceptible incurring 100% mortality. Mice infected by both exposure routes experienced weight loss, high viremia, and severe neuropathologic changes. The most significant histopathological findings occurred in the central nervous system where lesions represent an acute to subacute encephalitis/encephalomyelitis that is characterized by neuronal death, astrogliosis, microgliosis, scattered necrotic cellular debris, and inflammatory cell infiltrates. This model of ZIKV pathogenesis will be valuable for evaluating medical countermeasures and the pathogenic mechanisms of ZIKV because it allows immune responses to be elicited in immunologically competent mice with IFN I blockade only induced at the time of infection.

## Author Summary

Research addressing the severe clinical complications associated with ZIKV infection, including GBS and congenital ZIKV syndrome, are urgently needed. Key to this effort is development of well-characterized animal models that recapitulate human disease. Adult wild-type mice infected with ZIKV can develop viremia in some instances, but they do not emulate the disease associated with the severe congenital and adult neuropathology. Several groups have recently described type I or type II IFN-deficient murine models that are permissive for viral replication in several organs including the brain. The major limitation of these models is they utilize immunodeficient knockout mice lacking key components of the innate antiviral response. We describe the use of a lethal murine model for ZIKV where the innate response of immunocompetent mice is suppressed only at the time of infection. We show that the mice develop severe neurological disease similar to that previously demonstrated in mice deficient in the type I or II IFN response. Using this model, we provide a detailed description of the ZIKV-associated pathologic changes, which mirrors the neuropathogenic properties of ZIKV in humans. These studies provide a baseline for assessment of medical countermeasures that can prevent or treat such pathogenic effects caused by ZIKV infection.

## Introduction

Zika virus (ZIKV, *Flaviviridae*, *Flavivirus*) is an arthropod-borne virus (arbovirus) that is closely related to dengue, West Nile, Japanese encephalitis and yellow fever viruses [1,2]. ZIKV was first isolated in Uganda in 1947 from a febrile sentinel rhesus monkey in the Zika forest [3,4]. No significant outbreaks of ZIKV infection involving more than a few persons were detected until 2007, when ZIKV caused an explosive outbreak in Micronesia [5–8] where approximately 75% of the population on the island of Yap became infected during a four-month period [5]. In subsequent years, ZIKV continued to spread throughout Oceania [9–12]. In early 2015, ZIKV first emerged in the Western Hemisphere with an outbreak detected in Brazil [13,14]. The virus spread rapidly throughout Latin America and the Caribbean and within one year most countries in the region reported local transmission [15,16]. ZIKV is expected to continue to spread and imported cases from travelers returning from Latin America and the Caribbean have already been reported in several countries including the U.S. and Europe [15,17–19]. In fact, numerous locally acquired mosquito-borne cases have recently been reported in Florida.

Historically, infections with ZIKV are asymptomatic and have been associated with a self-limiting febrile illness with no long-term sequelae, but more severe complications have become apparent during the recent outbreaks in the South Pacific and Latin America. In particular, significant concern is growing about the association of ZIKV infection and the development of fetal abnormalities such as microcephaly. ZIKV was isolated from the brains and cerebrospinal fluid of neonates born with microcephaly and identified in the placental tissue of mothers who had symptoms consistent with ZIKV infection during pregnancy [20–22]. An additional concern is the association of ZIKV infection and Guillain-Barré syndrome (GBS). GBS is an autoimmune polyradiculoneuropathy that can result in weakness, paralysis, and death [23–25], and was first associated with ZIKV infection during the 2013–2014 outbreak in French Polynesia. Cases of a diffuse demyelinating disorder consistent with GBS that are temporally associated with ZIKV infection have been reported in Brazil, El Salvador, Colombia, and Venezuela [26]. As ZIKV continues to spread, so does concern about the association of ZIKV infection and the development of severe clinical complications. Therefore, the development of medical countermeasures for ZIKV is a high research priority.

Animal models are needed to better understand the pathogenic mechanisms of ZIKV and to evaluate candidate medical countermeasures. Early ZIKV mouse models have relied on the use of juvenile animals and/or intracerebral inoculations [3,4,27–35]. These initial studies suggest that in mice ZIKV can replicate and cause injury in cells of the CNS. In contrast, other animals to include cotton rats, guinea pigs, rabbits, and rhesus monkeys did not develop CNS disease even when infected by intracerebral inoculation [3]. In mice, neuronal degeneration and cellular infiltration were observed in regions of the spinal cord and brain [3]. Neuronal injury was also evident in the pathological evaluation of a human fetus infected *in utero* with ZIKV. Diffuse astrogliosis and activation of microglia were observed and damage extended to the brain stem and spinal cord [22]. Recently, mice deficient in the type I or type II interferon response developed severe neurological disease due to ZIKV infection [36–40]. ZIKV-infected *Ifnar1*^*-/-*^ mice (C57BL/6 background mice lacking the IFN α/β receptor) developed disease that was associated with high viral titers in the brain and spinal cord [39]. Similar results were described for A129 mice (129Sv/Ev background mice lacking the IFN α/β receptor), which were highly susceptible to ZIKV and developed neurological disease [36,38]. AG129 mice (129Sv/Ev background mice lacking the IFN α/β and γ receptors) were found to be more susceptible to ZIKV-induced disease compared to A129 mice [36]. Collectively, these efforts underscore the importance of innate immunity in modulating ZIKV infection and disease outcome.

The major limitation of these recently described ZIKV mouse models is that they utilize immunodeficient mice. These mouse models lack a key component of antiviral immunity which impairs comprehensive evaluation of medical countermeasures. In an attempt to produce infection models that do not rely upon knockout mice, several groups, including ours, have explored the temporal blockade of IFN-I in immune intact mice using polyclonal and monoclonal antibodies targeting either IFN-Is directly or the IFN-I receptor. The major advantage of this approach is that it allows immune responses to be elicited in immunologically competent mice with IFN I blockade only induced at the time of infection. A murine non-cell depleting monoclonal antibody (MAb) that efficiently targets the IFNAR-1 subunit of the mouse IFN-α/β receptor (MAb-5A3) was developed and shown to prevent type I IFN-induced intracellular signaling *in vitro* and to inhibit antiviral, antimicrobial, and antitumor responses in mice [41]. MAb-5A3 has been used to explore the role of IFN-I in the infection and pathogenesis of several viruses including West Nile virus (WNV) [42], lymphocytic choriomeningitis virus, and vesicular stomatitis virus (VSV) [41]. For VSV and WNV, treatment of mice with this antibody results in a severe and lethal infection model similar to that produced in IFN-I receptor knockout mice. Here, we describe the use of MAb-5A3 antibody to block IFN-I signaling in immune intact, wild-type mice at the time of ZIKV infection. We demonstrate that these mice develop severe ZIKV-mediated disease accompanied by significant neuroinflammation and mortality when infected by multiple exposure routes. While we were completing this study, another group also reported the use of this system for ZIKV studies [39,43]. Although in the first report, these authors were unable to demonstrate ZIKV lethality, in the second study, they did find lethality using an African lineage strain that was derived from a brain homogenate by passage of the virus in *Rag1*^*-/-*^ mice. Our report not only expands on those findings, but also provides the first comprehensive description of the pathologic changes associated with ZIKV infection using this model. This model of ZIKV pathogenesis will be valuable for evaluating medical countermeasures because it allows an immune response to be elicited in immunocompetent mice and infection is enhanced at the time of virus challenge.

## Methods

### Ethics statement

This work was supported by an approved USAMRIID IACUC animal research protocol. Research was conducted under an IACUC approved protocol in compliance with the Animal Welfare Act, PHS Policy, and other Federal statutes and regulations relating to animals and experiments involving animals. The facility where this research was conducted is accredited by the Association for Assessment and Accreditation of Laboratory Animal Care, International and adheres to principles stated in the Guide for the Care and Use of Laboratory Animals, National Research Council, 2011. Approved USAMRIID animal research protocols undergo an annual review every year. Animals are cared for by a large staff of highly qualified veterinarians, veterinary technicians, and animal caretakers. All personnel caring for and working with animals at USAMRIID have substantial training to ensure only the highest quality animal care and use. Humane endpoints were used during all studies and mice were humanely euthanized when moribund according to an endpoint score sheet. Mice were euthanized by terminal exsanguination or CO_2_ exposure using compressed CO_2_ gas followed by cervical dislocation. However, even with multiple observations per day, some animals died as a direct result of the infection.

### Virus and pathogenesis study design

ZIKV strain DAK AR D 41525 isolated in 1984 from *Aedes africanus* mosquitoes in Senegal and was obtained from the World Reference Center for Emerging Viruses and Arboviruses (R. Tesh, University of Texas Medical Branch) where it was amplified once in AP61 and C6/36 cells, and two times in Vero cells prior to our receipt. We then amplified the virus once more in Vero cells (ATCC, CCL-81) prior to use in this study and sequenced [44]. Female C57BL/6 mice (n = 10/group; Jackson Laboratories) five weeks of age were injected IP with a total of 3.0 mg (2.0 mg first dose, 0.5 mg subsequent doses) of MAb-5A3 (produced by Leinco Technologies, St. Louis, MO) [41,45] or PBS on day -1, day +1, and day 4. A prior study characterizing this antibody indicated that a large bolus is needed to saturate the IFNAR-1 receptor pool and the half-life of a 2.0 mg injected dose is 5.2 days [41]. On day 0, mice were infected with 6.4 log_10_ PFU of ZIKV strain DAK AR D 41525 by the SC (in between the shoulder blades) or IP exposure route in a total volume of 200 μL. Mice were monitored for signs of disease and bled on day 4 post-infection (PI) or when euthanized to evaluate viremia. A cohort of Mab-5A3-treated, uninfected control mice (n = 3) was included for histopathology assessment. These control mice were treated with the antibody as described above and euthanized on day 10.

### qRT-PCR assay

Mouse serum samples were inactivated using a 3:1 ratio of TRIzol LS Reagent (Thermo Fisher Scientific, Waltham, MA). Tissues were homogenized in 1X Minimum Essential Medium with Earle’s Salts and L-glutamine (MEM) with 1% penicillin/streptomycin and 5% heat-inactivated fetal bovine serum (FBS-HI) using a gentleMACS dissociator (Miltenyi Biotec, San Diego, CA) followed by centrifugation at 10,000 x g for 10 minutes and the supernatant was stored at -80°C until further evaluation. Supernatant was inactivated using a 3:1 ratio of TRIzol LS. Total nucleic acid from all samples was purified using the EZ1 Virus Mini Kit v 2.0 (Qiagen, Valencia, CA) and the EZ1 Advanced XL robot (Qiagen) according to the manufacturer’s recommendations. Samples were eluted in 60μL. Viral load was determined using a real-time RT-PCR assay specific to the 5’-untranslated region of ZIKV. Specific amplification detection was accomplished using a forward primer (5’-GARTCAGACTGCGACAGTTCGA), reverse primer (5’-CCAAATCCAAATTAAACCTGTTGA), and probe (5’-ACTGTTGTTAGCTCTCGC–MGBNFQ). A standard curve was generated using serial dilutions of the challenge virus having PFU/mL titers determined by plaque assay. Five μL of extracted nucleic acid were run in triplicate on the LightCycler 480 (Roche Diagnostics, Inc., Indianapolis, IN) using SuperScript One-Step RT-PCR (Thermo Fisher Scientific), and samples were considered negative if the cycle of quantification (Cq) was greater than 40 cycles. This Cq cutoff value was selected because when Cq's are greater than 40 cycles, you are outside of the linear dynamic range of real-time PCR, and thus, can negatively impact data reproducibility. The virus titers were calculated using the standard curve and the LightCycler 480 software, and the final PFU equivalents/mL (PFUe/mL) calculations were determined based on the sample input volumes and the upfront sample dilutions.

### Plaque assay

Vero cells were plated at 3x10^5^ cells/well in a six-well plate and incubated overnight at 37°C, 5% CO_2_. Serial dilutions of samples were made in 1X MEM with 1% penicillin/streptomycin and 5% heat-inactivated FBS. Uninfected-control and serially-diluted samples were incubated with the Vero cells for one hour at 37°C, 5% CO_2_ for virus adsorption. The inoculum was removed and a 1:1 mixture of 0.8% (w/v) Seaplaque agarose and 2X Basal Medium Eagle with Earle’s Salts (EBME) solution containing 2X EBME, 10% FBS-HI, 2% penicillin/streptomycin, 50 μg/mL gentamicin, and 2.5 μg/mL Fungizone/Amphotericin B was added. After addition, the 0.4% Seaplaque agarose/2X EBME overlay was incubated at room temperature for 30 minutes to allow the overlay to solidify. Vero cells were incubated with the overlay at 37°C, 5% CO_2_ for five days before the overlay was removed. Cells were fixed and plaques were visualized by a 20 minute addition of 10% formalin with 50% Crystal Violet solution followed by a wash with water.

### Histology

A necropsy was completed to collect the spleen, liver, head (to include brain), heart, kidney and spinal cord. All collected tissues were immersion fixed in 10% neutral buffered formalin for at least 2 days. The tissues were trimmed and processed according to standard protocols [46]. Histology sections were cut at 5 to 6 μM on a rotary microtome, mounted onto glass slides, and stained with hematoxylin and eosin (HE). Unblinded histological examination was performed by a board-certified veterinary pathologist.

### ZIKV RNA *in situ* hybridization (ISH)

*In situ* hybridization was performed using RNAscope 2.5 HD RED kit according to the manufacturer’s recommendations (Advanced Cell Diagnostics, Hayward, CA). Briefly, 20 ZZ probes set targeting the 1550–2456 fragment of the ZIKV polyprotein gene [47] were synthesized. After deparaffinization and peroxidase blocking, the sections were heated in antigen retrieval buffer and then were digested by proteinase. The sections were covered with ISH probes and incubated at 40°C in a hybridization oven for two hours. They were rinsed and the ISH signal was amplified by applying Pre-amplifier and Amplifier conjugated with HRP. A red substrate-chromogen solution was applied for 10 minutes at room temperature. The slides were further stained with hematoxylin, air dried, and mounted.

### Infrared and immunofluorescent imaging

Formalin-fixed, paraffin-embedded mouse brain sections on slides were deparaffinized in xyless and rehydrated through graded ethanol (100%, 95%, 90%, and 70%). Antigen was retrieved by citric acid-based antigen unmasking solution (Vector Laboratories) during 10 minute boiling. After three washes with PBS (pH 7.4), the sections were blocked with 10% normal donkey serum in PBS-tween (0.1%; PBS-T) for one hour at room temperature. The sections were incubated with primary antibodies, goat anti-Iba1 (3 μl/mL; Novus Biotechnology) and Rabbit anti-GFAP (1:5000; Abcam), diluted in 10% normal donkey serum in PBS-T overnight at 4°C. After washing in PBS-T (3x5 min), the sections were incubated for 2 h at room temperature with secondary antibodies diluted in 10% normal donkey serum in PBS-T. For standard immunofluorescence, the secondary antibodies were donkey anti-goat Alexa Fluor 488 (1:300; Invitrogen) and donkey anti-rabbit Rhodamine-Red-X (1:200; Jackson ImmunoResearch). The nuclei were stained with Hoecht’s. For infrared analysis, the secondary antibodies were donkey anti-goat IRDye 680RD (1:1500; Li-cor Biosciences) and donkey anti-rabbit IRDye 800CW (1:1500; Li-cor Biosciences). The sections were subsequently washed in PBS-T (3x10 min), PBS (3x5 min), and water (2x5 minutes). For immunofluorescence, the sections were cover slipped with Fluoromount-G (SouthernBiotechnology). For double-fluorescence labeling, primary rabbit anti-Zika Envelope (E) glycoprotein (1:400, IBT Bioservices) and mouse anti-alpha-Smooth Muscle Actin (1:200 Clone 1A4, R&D Systems) and secondary Alexa Fluor 488 conjugated goat anti-rabbit and Alexa Fluor 561 conjugated goat anti-mouse antibody were used. Rabbit IgG isotype (1:500; cat# MA5-16384, ThermoFisher Scientific) was used as an immunofluorescence staining control. The nuclei were stained with 4′,6-diamidino-2-phenylindole (DAPI). Images were captured on a Zeiss LSM 780 confocal system and processed with Zen 2011 confocal, Photoshop, or ImageJ software. Sections for infrared analysis were air-dried overnight. A Licor-Odyssey CLx (Li-cor Biosciences) scanned sections at 21 μm/pixel resolution. The average intensities of GFAP and Iba1 on each slide were obtained from fields-of-interest draw around each section with the Li-cor-Odyssey analysis software on at least two sections per slide and three slides per brain were scanned. Negative control staining, for which the primary antibodies were omitted, showed no detectable labeling in immunofluorescence or infrared imaging.

### Statistical analysis

Survival analysis was completed by Kaplan Meier estimate. A one-way ANOVA by Kruskal-Wallis test with Dunn’s multiple comparisons was used to analyze differences in viremia. An unpaired t-test was used to compare Iba1 and GFPA expression. SAS version 9.1.3 (SAS Institute Inc., Cary NC) was used for all analyses.

## Results

### ZIKV causes high mortality in immunocompetent mice when IFN-I signaling is blocked

**Fig 1:**
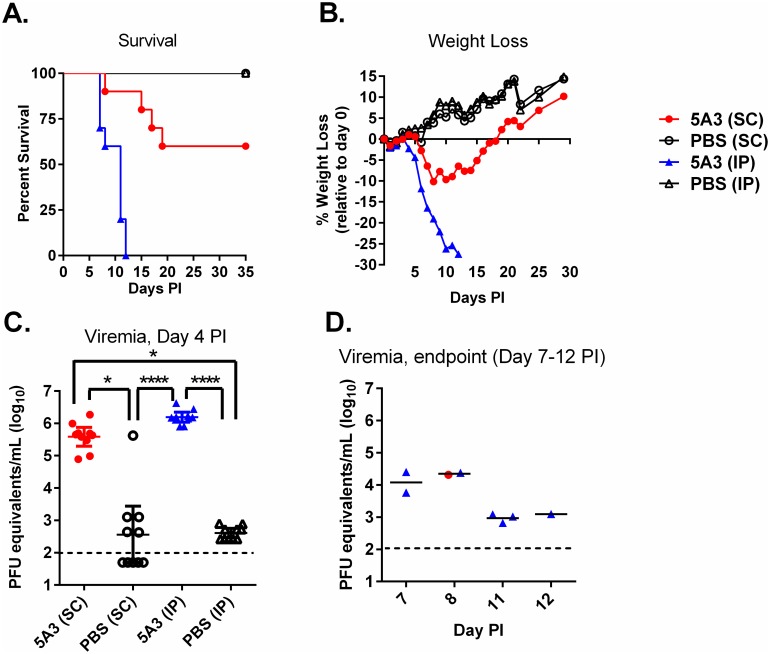
Wild-type Mice Treated with an IFNAR1-Blocking MAb are Susceptible to ZIKV. Five week old wild-type mice were treated with an IFNAR1-blocking MAb or PBS by intraperitoneal (IP) injection and then exposed to 6 log_10_ of ZIKV strain DAK AR D 41525 subcutaneously (SC) or IP. Mice were monitored for survival (A) and weight loss shown as percent change in baseline prior to infection (B). ZIKV RNA in serum was determined on day 4 post-infection (PI) (C) and when the mice were euthanized (D) by qRT-PCR. Data are shown as PFU equivalents (PFUe) per milliliter after normalization to a standard curve. Symbols represent the individual mice, the line represents the geometric mean, and the error bars represent the 95% confidence interval. The dotted line represents the assay limit of detection. Statistically significant differences are denoted by an asterisk (*p < 0.05; *p < 0.0001).

Immunocompetent mice were treated with MAb-5A3 to block IFN-I signaling or PBS prior to and after challenge with 6 log_10_ PFU of ZIKV given by IP or SC injection. All of the PBS-treated mice challenged with ZIKV by either route survived and no apparent signs of disease, including weight loss were observed (Fig 1A and 1B). Mice exposed to ZIKV by the IP route and treated with MAb-5A3 began to succumb or were euthanized on day 7 PI at which time the mice presented with ruffled fur, hunched posture, and were poorly responsive. On day 8 PI, a mouse exposed SC and another mouse exposed IP exhibited right-side, hind-limb paralysis and were euthanized. Mice treated with MAb-5A3 and exposed to ZIKV by the IP route continued to succumb or were euthanized through day 12 PI where 100% (n = 10/10) mortality was observed. MAb-5A3-treated mice challenged with ZIKV by the SC route continued to succumb through day 19 PI and resulted in 40% (n = 4/10) mortality. The mean times-to-death (MTD) for mice exposed IP was 9.7 days and for mice exposed SC was 14.75 days, which were significantly different (P<0.0001). Weight loss corresponded with survival for both challenge routes for mice treated with MAb-5A3. Mice treated with MAb-5A3 and exposed to ZIKV IP or SC began to lose weight on day 4 PI and 6 PI, respectively. Weight loss continued for MAb-5A3treated mice exposed to ZIKV IP through day 12 PI when all mice had succumbed or were euthanized. MAb-5A3-treated mice exposed to ZIKV SC continued to lose weight until day 8 PI and then slowly began to regain weight and return to baseline values relative to day 0 PI by day 19 PI when mortality was no longer observed. However, we cannot determine if weight loss occurred only in mice succumbing from SC ZIKV infection because mice were weighed by group and not individually. These findings indicated that ZIKV can cause high mortality in mice with intact immune systems when IFN-I is blocked and that IP exposure was more lethal than SC exposure.

### Viral titers in sera and tissues of ZIKV-infected mice

**Fig 2:**
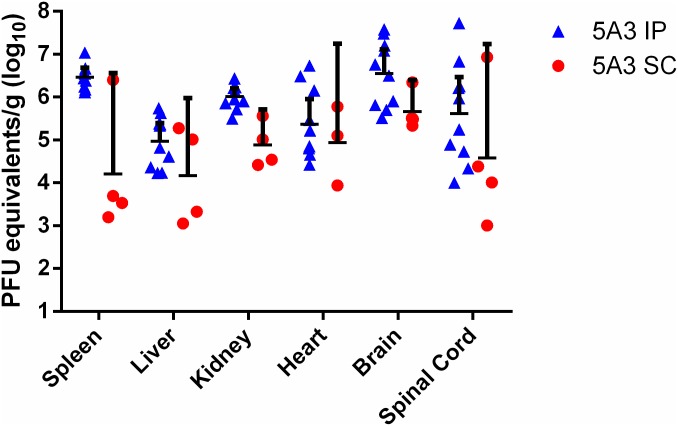
Viral titers of ZIKV in Wild-type Mice Treated with an IFNAR1-Blocking MAb. Five week old wild-type mice were treated with an IFNAR1-blocking MAb or PBS by intraperitoneal (IP) injection and then exposed to 6 log_10_ of ZIKV strain DAK AR D 41525 subcutaneously (SC) or IP. When mice succumbed or were euthanized, tissues were collected, weighed, homogenized, and analyzed by qRT-PCR. Data are shown as PFU equivalents (PFUe) per gram (g) after normalization to a standard curve. Symbols represent the individual mice, the line represents the geometric mean, and the error bars represent the 95% confidence interval.

The viral titers in the sera were determined on day 4 PI and when mice were euthanized (Fig 1C and 1D). All mice treated with MAb-5A3 and exposed to ZIKV developed viremia, however these levels were slightly (but not significantly) higher on average (0.6 log_10_ PFUe/mL higher) in IP exposed animals versus SC exposed animals. While all PBS-treated mice exposed to ZIKV by IP injection developed viremia, it was significantly (p<0.0001) lower on average (3.6 log_10_ PFUe/mL lower) compared to MAb-5A3-treated mice exposed to ZIKV IP. Most mice treated with PBS and exposed SC to ZIKV had viremia below the limit of detection for our assay (5/10), however, one mouse had a titer of >5 log_10_ PFUe/ml. There was no significant difference in the viremia of mice treated with PBS and exposed SC vs. IP. In addition to analyzing day 4 viremia in all mice, we also measured the viremia in animals succumbing to infection. At the time of euthanasia, viremia was generally lower compared to levels observed on day 4 PI. At time of euthanasia or death, unperfused tissues were collected and viral titers in the liver, spleen, heart, kidney, brain, and spinal cord were measured by qRT-PCR (Fig 2). Virus was detected in all tissues collected in mice treated with MAb-5A3 and exposed IP and SC to ZIKV. The tissues were collected from moribund mice on different days PI, which does not warrant direct comparison of the results by statistics. The viral titers here represent the detection of viral RNA and not infectious virus, so we completed plaque assays on a subset of samples and confirmed the presence of infectious virus in the brain (a key target tissue in this study) where 3.9 or 3.8 PFU/g was detected in antibody treated mice exposed IP or SC to ZIKV, respectively. Collectively, these findings demonstrated that ZIKV infection in mice with IFN-I blockade results in viremia and tissue titers. However, these mice were not perfused so some of the virus detected in the tissues may be from the blood.

### ZIKV infection targets the CNS

**Fig 3:**
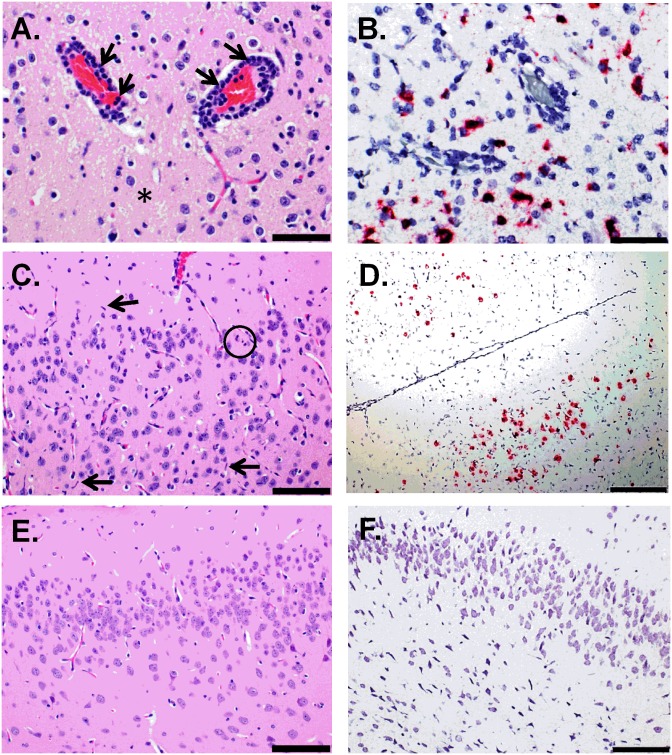
Histologic and ISH Findings in the Cerebrum of ZIKV-Infected Wild-type Mice Treated with an IFNAR1-Blocking MAb and Uninfected Control Mice. (A-B) The cerebrum of a mouse exposed to ZIKV intraperitoneally (IP) that succumbed on day 7 post-infection (PI). (A) Representative hematoxylin and eosin staining showed perivascular cuffing of vessels with mononuclear cells and few neutrophils (indicated by arrows). Within the adjacent, vacuolated neuropil there are few neutrophils admixed with scattered necrotic cellular debris and activated microglial cells (representative area indicated by an asterisk); scale bar represents 50 μm. (B) Representative ISH staining demonstrating that ZIKV RNA is scattered throughout the same area; scale bar represents 50 μm. (C-D) The cerebrum of a mouse exposed to ZIKV IP that was euthanized on day 11 PI. (C) Representative hematoxylin and eosin staining showed the neuropil contains scattered necrotic cellular debris (representative area indicated by a circle) and activated microglia (representative cells indicated by arrows); scale bar represents 100 μm. (D) Representative ISH staining demonstrating that ZIKV RNA is detected throughout the same region; scale bar represents 200 μm. (E) Representative hematoxylin and eosin staining in the cerebrum of an uninfected control mouse; scale bar represents 100 μm. (F) Representative ISH staining demonstrating no ZIKV RNA is detected in the cerebrum of an uninfected control mouse; scale bar represents 100 μm. These findings are from one independent experiment where a total of 10 ZIKV-infected mouse brains (3 uninfected controls) were analyzed by an unblinded, board-certified veterinary pathologist.

We completed ISH and IFA coupled with histopathological analysis in tissues from ZIKV-infected mice that succumbed or were euthanized due to severe disease. Significant histopathological changes occurred in the CNS of all ZIKV-infected animals treated with the IFN-I blocking MAb-5A3 (Fig 3, S1 Table). The most notable microscopic lesions attributable to ZIKV infection in these mice included evidence of minimal to mild inflammation and necrosis in the brain and spinal cord of all animals that succumbed on days 7 and 8, and to a lesser extent, in at least 5 of the 6 animals that succumbed between days 11 and 12. The presence and /or extent of CNS lesions were difficult to ascertain in a few of the animals (not included in the assessment) due to artifactual damage to the tissue (S1 Table) and autolysis precluded the assessment of three animals that succumbed after day 12 PI and were removed from the histopathology results. Findings in the CNS included perivascular infiltrates of mononuclear cells (“perivascular cuffing") and multifocal to diffuse gliosis with activated microglia (Fig 3A). ZIKV RNA was frequently detected in the same regions by ISH (Fig 3B). Perivascular cuffing was a more consistent finding in mice that succumbed earlier (days 7–8) than in those that died or were euthanized later in the course of disease. In the sections examined, 5 of 5 animals from days 7–8, and 2 of 5 animals from days 11–12 exhibited perivascular cuffing in the brain; 4 of 4 animals from days 7–8, but none from days 11–12 exhibited perivascular cuffing in the spinal cord. In most animals, the perivascular cuffing was subtle, consisting of few mononuclear inflammatory cells; in 2 animals exposed to ZIKV IP that succumbed or were euthanized on day 7 PI, few neutrophils were admixed with the mononuclear cells. Microgliosis was a fairly consistent finding that was observed in the examined CNS sections of all animals from days 7 through 12. The severity was mild to occasionally moderate in animals that succumbed on days 7–8; whereas, microgliosis was minimal to occasionally mild in animals that succumbed on days 11–12 (Fig 3C).

**Fig 4:**
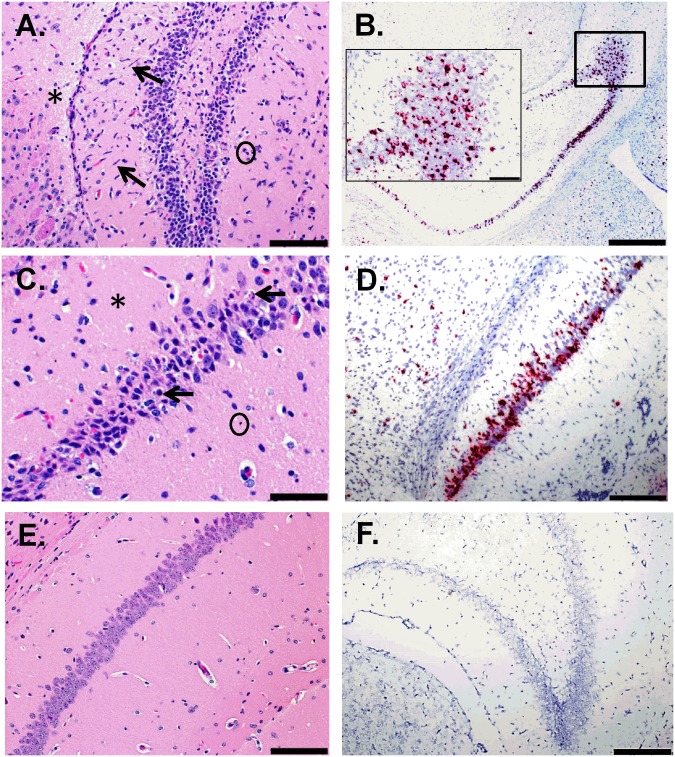
Histologic and ISH Findings in the Hippocampus of ZIKV-Infected Wild-type Mice Treated with an IFNAR1-Blocking MAb and Uninfected Control Mice. (A) The hippocampus of a mouse exposed to ZIKV intraperitoneally (IP) that was euthanized on day 11 post-infection (PI). Representative hematoxylin and eosin staining showed neuropil vacuolation (representative area indicated with an asterisk), microgliosis (representative cells indicated by arrows), and necrotic cellular debris (representative area indicated with a circle); scale bar represents 100 μm. (B) The hippocampus of a mouse exposed to ZIKV IP that was euthanized on day 7 PI. The representative ISH staining demonstrates massive ZIKV infection of the brain; scale bar represents 500 μm (inset picture scale bar represents 100 μm). (C-D) The hippocampus of a mouse exposed to ZIKV IP that was euthanized on day 7 PI. (C) The representative hematoxylin and eosin staining demonstrates that pyramidal neurons are often necrotic (representative cells indicated by arrows) and the adjacent vacuolated neuropil (representative area indicated with an asterisk) contains necrotic scattered cellular debris (representative area indicated by a circle); scale bar represents 50 μm. (D) Representative ISH staining demonstrating that ZIKV RNA is detected in the same region; scale bar represents 200 μm. (E) Representative hematoxylin and eosin staining in the hippocampus of an uninfected control mouse; scale bar represents 100 μm. (F) Representative ISH staining demonstrating no ZIKV RNA is detected in the hippocampus of an uninfected control mouse; scale bar represents 200 μm. These findings are from one independent experiment where a total of 10 ZIKV-infected mouse brains (3 uninfected controls) were analyzed by an unblinded, board-certified veterinary pathologist.

Additional findings in the CNS included multifocal areas of neuropil vacuolation (edema) and scattered necrotic cellular debris, most notably in the cerebrum (Fig 3C), hippocampus (Fig 4A) and thalamus. Again, ZIKV RNA was detected by ISH in the same regions as these histopathological changes (Figs 3D and 4B), which is in contrast to the uninfected control mice (Figs 3E, 3F, 4E and 4F). Multifocal areas of edema occurred in 5/5 animals from days 7–8, and in 2/5 animals from days 11–12. Edema frequently surrounded blood vessels and was most prevalent in areas with the most abundant gliosis and neuronal necrosis. Scattered necrotic cellular debris often occurred adjacent to blood vessels and was observed in the brain of 5/5 animals from days 7–8, and in 5/5 animals from days 11–12. Although necrotic debris was often observed in multiple CNS sections, it was most frequently observed in the hippocampus, thalamus, cerebrum and less so in the cerebellum, pons and spinal cord. It is difficult to determine the cell type from which this necrotic debris originated—possibilities include neurons, resident glia, or infiltrating leukocytes.

An additional notable finding in the CNS was neuronal necrosis, characterized by hypereosinophilic neurons and pyknosis, karyolysis, and replacement of neurons with necrotic debris. Neuronal necrosis occurred most frequently and extensively in the hippocampal pyramidal and granule layers and less frequently in the thalamus, cerebrum, and least frequently in the cerebellum, pons and spinal cord (Fig 4C). Once again, RNA was consistently detected in corresponding regions of the brain by ISH (Fig 4D). In the sections examined, neuronal necrosis was observed in the brain of 4/5 animals from days 7–8 and 3/5 animals from day 11–12. Neuronal necrosis was also observed in the spinal cord of 4/4 animals examined from days 7–8 and 2/3 animals examined from day 11–12. Less frequently there was neuronal degeneration and satellitosis. Another, although less consistent finding, was minimal neutrophilic infiltrates scattered within the brain parenchyma, which was observed in only 2/5 animals from days 7–8, and 3/5 animals from days 11–12.

**Fig 5:**
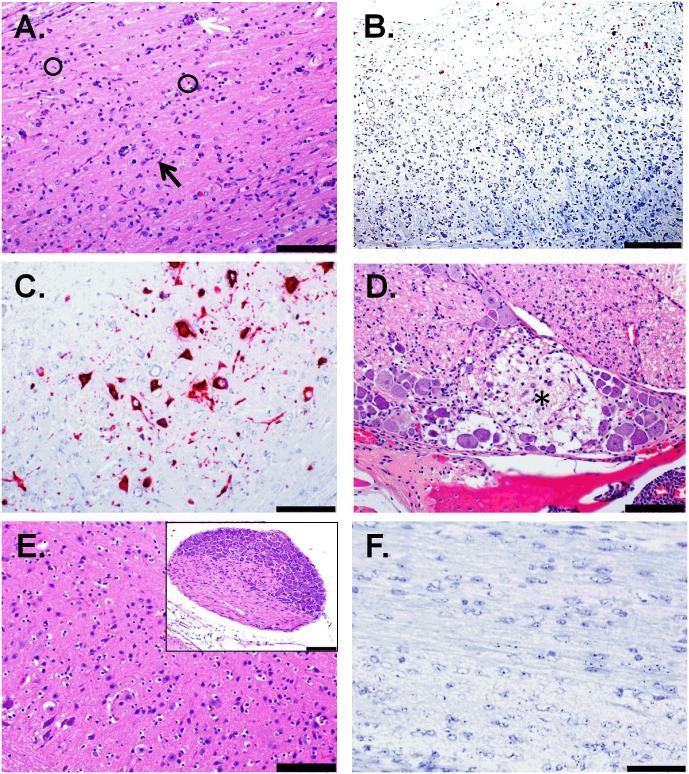
Histologic and ISH Findings in the spinal cord of ZIKV-Infected Wild-type Mice Treated with an IFNAR1-Blocking MAb and Uninfected Control Mice. (A-B) The spinal cord of a mouse that was exposed to ZIKV subcutaneously (SC) and euthanized on day 8 post-infection (PI). (A) Representative hematoxylin and eosin staining showed there is scattered necrotic cellular debris (representative areas indicated by circles), gliosis (representative cells indicated by black arrow), and perivascular cuffing (indicated by white arrow); scale bar represents 100 μm. (B) Representative ISH staining demonstrating that ZIKV RNA is detected throughout the same region; scale bar represents 200 μm. (C) ISH staining demonstrating massive ZIKV infection in the spinal cord of a mouse that was exposed to ZIKV intraperitoneally (IP) and was euthanized on day 7 PI; scale bar represents 100 μm. (D) Hematoxylin and eosin staining of spinal ganglion demonstrates a focal area of necrosis (indicated by an asterisk) with adjacent gliosis and few infiltrating neutrophils in a mouse exposed to ZIKV IP that succumbed on day 7 PI; scale bar represents 100 μm. (E) Representative hematoxylin and eosin staining in the spinal cord of an uninfected control mouse; scale bar represents 100 μm. Inset is a spinal ganglion from an uninfected control mouse; scale bar represents 100 μm. (F) Representative ISH staining demonstrating no ZIKV RNA is detected in the spinal cord of an uninfected control mouse; scale bar represents 200 μm. These findings are from one independent experiment where a total of 6 ZIKV-infected mouse spinal cords (3 uninfected controls) were analyzed by an unblinded, board-certified veterinary pathologist.

Histopathological analysis of the spinal cord showed evidence of one or more of the following: gliosis, neuronal satellitosis, and perivascular inflammatory infiltrates, in all mice observed (Fig 5A). The detection of ZIKV RNA by ISH in the spinal cord suggests that these lesions are also due to infection (Fig 5B and 5C). In general, cells in the spinal cord were only occasionally observed to be positive for ZIKV RNA by ISH; however, in some animals the spinal cord was more severely affected, and massive ZIKV infection was detected by ISH in some animals as depicted in Fig 5C. Additionally, a focally extensive area of necrosis affecting a spinal ganglion was observed in a mouse exposed to ZIKV IP that succumbed on day 7 PI (Fig 5D). The noted pathologic changes and ISH findings in the spinal cord of ZIKV-infected mice is in contrast to what was observed in uninfected control mice (Fig 5E and 5F).

**Fig 6:**
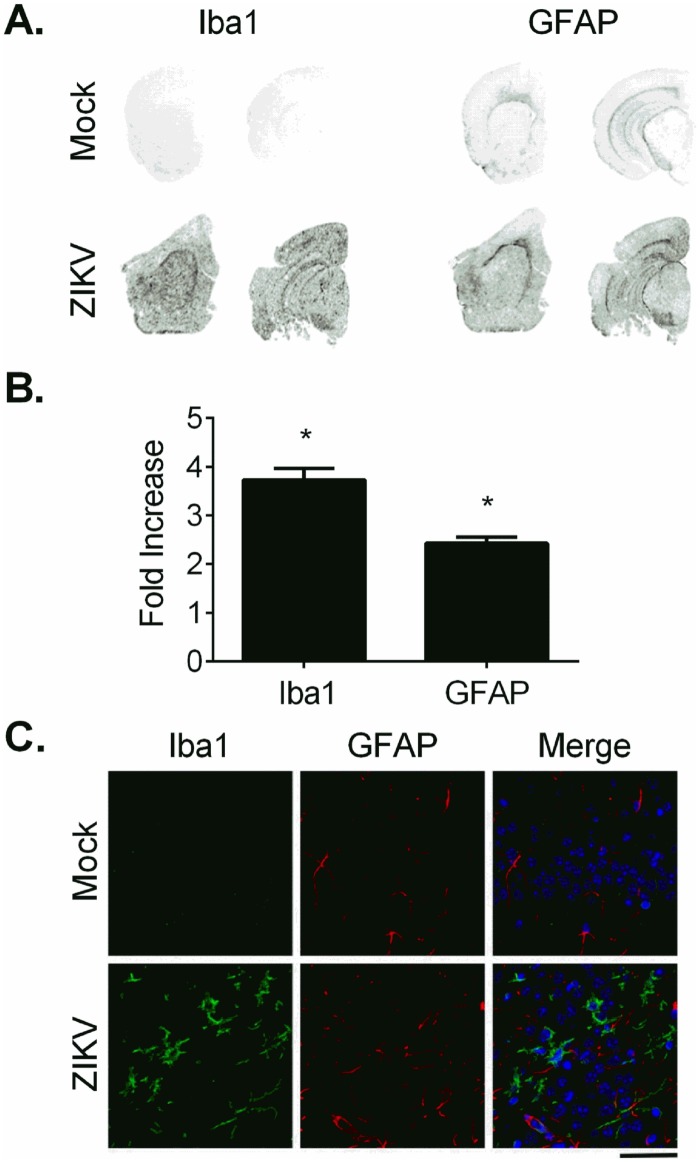
Iba1 and GFAP are Increased in the Brains of ZIKV-Infected Wild-type Mice Treated with an IFNAR1-Blocking MAb. (A-B) Brain sections from mice treated with IFNAR1-blocking MAb and infected with ZIKV or mock infected with PBS were imaged and analyzed by infrared imaging. Representative images showed increased Iba1 and GFAP staining in brain sections from ZIKV-infected mice compared to PBS-inoculated mice. (B) The average infrared intensity, expressed as fold increase relative to PBS, was significantly increased in ZIKV-infected mice compared to PBS-inoculated mice for both Iba1 (PBS: 0.9929 ± 0.1614, n = 5; ZIKV: 3.725 ± 0.2415, n = 10; *p < 0.0001, t-test) and GFAP (PBS: 1.000 ± 0.1022, n = 5; ZIKV: 2.429 ± 0.1252, n = 10; *p < 0.0001, t-test). (C) Representative images of Iba1 and GFAP immunofluorescent labeling of Iba1 (green) and GFAP (red) in brain sections of mock- or ZIKV-infected mice. Scale bar represents 50 μm.

It has been known that both ionized calcium binding adaptor molecule 1 (Iba1) expressed by microglia and glial fibrillary acidic protein (GFAP) expressed by astrocytes are upregulated in activated microglia and astrocytes, respectively, during neuroinflammation [48,49]. Infrared and immunofluorescent imaging was used to assess the effect of ZIKV on neuroinflammation by detecting the expression of Iba1 and GFAP in brain sections from 10 of the mice that succumbed to ZIKV infection (brains from four of the mice were not analyzed due to artifactual damage). Compared to brains of control mice treated with IFNAR1-blocking MAb-5A3, Iba1 and GFAP levels were significantly increased in the brains of ZIKV-infected mice (Fig 6A and 6B) and these findings were confirmed by immunofluorescent labeling of Iba1 and GFAP in these brains (Fig 6C). Collectively, the results suggest that in our model, CNS infection by ZIKV results in significant neuroinflammation.

### Additional pathologic changes induced by ZIKV infection

**Fig 7:**
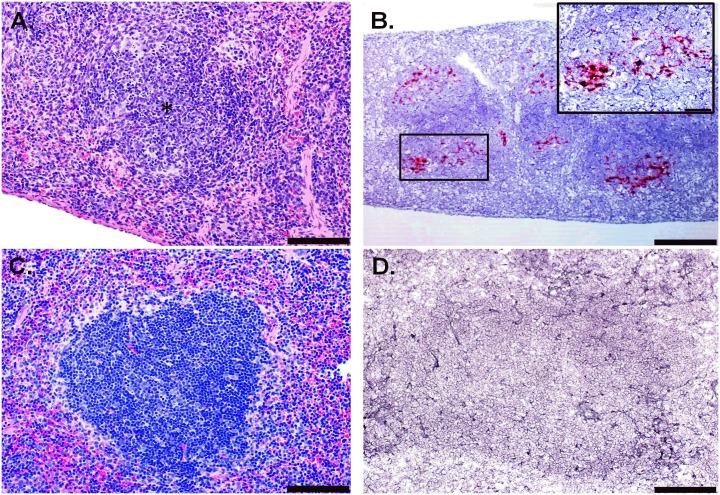
Histologic and ISH Findings in the Spleen of ZIKV-Infected Wild-type Mice Treated with an IFNAR1-Blocking MAb and Uninfected Control Mice. (A) Representative hematoxylin and eosin staining showed lymphocytolysis within the follicles (representative area indicated with an asterisk) in the spleen of a mouse exposed to ZIKV intraperitoneally (IP) that was euthanized on day 11 post-infection (PI); scale bar represents 100 μm. (B) Representative ISH staining demonstrating that multifocally, ZIKV RNA is detected in the lymphoid follicles in the spleen of a mouse exposed to ZIKV IP that was euthanized on day 11 PI; scale bar represents 200 μM (inset scale bar represents 50 μm). (C) Representative hematoxylin and eosin staining in the spleen of an uninfected control mouse; scale bar represents 100 μm. (D) Representative ISH staining demonstrating no ZIKV RNA is detected in the spleen of an uninfected control mouse; scale bar represents 100 μm. These findings are from one independent experiment where a total of 8 ZIKV-infected mouse spleens (3 uninfected controls) were analyzed by an unblinded, board-certified veterinary pathologist.

Other significant findings outside of the CNS include necrotic and/or apoptotic cellular debris within the splenic lymphoid follicles (white pulp) interpreted as lymphocytolysis (Fig 7A). This was a fairly consistent finding in these mice, with similar lesions being observed in all (8/8) spleens examined (S1 Table). The severity of the lymphocytolysis varied from mild in the earliest stages (3/3 animals from day 7) to minimal in all subsequent animals. Additionally, several animals (2 from day 7, 1 from day 8 and 1 from day 11) exhibited minimal lymphoid hyperplasia in the spleen, presumably in response to ZIKV infection. ZIKV RNA was also consistently detected by ISH in cells in the white pulp of the spleens from these animals (Fig 7B), which is in contrast to the histopathology and ISH findings observed in uninfected control mice (Fig 7C and 7D). No significant histopathological findings were observed in the liver, kidney, and heart of antibody treated, ZIKV-infected mice; however, ISH staining was observed in the smooth muscle cells within the tunica media of blood vessels in the kidney and heart, which was not observed in uninfected control mice (S1 Fig). IFA confirmed the presence of ZIKV in the smooth muscle of blood vessels in the kidney, which was not observed in uninfected control mice or by isotype control antibody staining (S1 Fig). The lack of concurrent microscopic evidence of tissue injury despite the presence of ZIKV in these mice suggests that, although virus is present in the smooth muscle, no direct damage to these cells is occurring; however, further studies are needed to fully elucidate the significance of ZIKV presence in smooth muscle of these blood vessels. Also, it is unclear why ZIKV appears to be present in the tunica media of vessels of these two organs but not in other organs examined.

Additional findings of interest outside the CNS include the observation of degeneration, inflammation, and less consistently, regeneration, of skeletal muscles of the head and vertebral column. Myocyte degeneration, inflammation, and nuclear rowing were evident by hematoxylin and eosin staining (S2 Fig). However, ZIKV was not detected in the skeletal muscle by ISH when the mice were moribund. Interestingly, ZIKV RNA was detected in a small subset of mice (n = 5) from an additional independent study where the mice were euthanized on day 3 PI (S2 Fig). ZIKV RNA was not detected by ISH in uninfected control mice where the histopathology appears normal (S2 Fig). It appears that ZIKV RNA is present in the skeletal muscle before mice become moribund, but is then cleared at later time points and inflammation persists. Inflammation and degeneration was a fairly common finding, and was noted in at least one anatomic site from all 11 animals examined. Regeneration was observed less frequently, with minimal regeneration occurring in 8 animals. Although lesions were observed in skeletal muscles from multiple anatomic sites, no similar lesions were observed in cardiac muscle. The skeletal muscle observed in this study was limited to the head and vertebral column regions. Future investigations should include examination of the skeletal muscles from the rear limbs, particularly since hind limb paralysis was observed in two of the mice.

## Discussion

The unexpectedly frequent and severe clinical complications of ZIKV infection, including GBS and congenital ZIKV syndrome, have prompted intense research on host-virus interactions. Key to these efforts is the development of well-characterized animal models that recapitulate human disease. Adult wild-type mice infected with ZIKV can develop viremia in some instances [39,50], but do not reflect the severe neurological disease seen in humans. However, transplacental and vaginal infection has been described in wild-type mice [51,52]. Knockout mice, deficient in type I or type II IFN responses, are permissive for viral replication in several organs including the brain [36–40]; however, infection in these mice does not provide an adequate means to test the efficacy of medical countermeasures or to study pathogenic events after infection. For example, in addition to its role in controlling viral infection through antiviral gene induction, type I IFN plays a role in priming of B and T cell responses [reviewed in [53,54]. Therefore, a more immunologically competent mouse model is essential for evaluating vaccines and treatments for ZIKV. The major advantage of this approach is that it allows immune responses to be elicited in immunologically competent mice with IFN I blockade only induced at the time of infection.

We developed and characterized a partially immunocompetent murine model of ZIKV infection replicating the pathologic changes noted in genetically modified mice through antibody blockade of the type I IFN receptor. We are using this model to evaluate vaccines and therapeutics and as reported herein, have used the model to establish a baseline of ZIKV pathogenesis. As we were completing our pathogenesis study, another group reported a similar model. In these studies, a non-lethal model of ZIKV infection was described in wild-type mice injected IP with 1 mg or 2 mg of the same IFN-1 blocking antibody that we used and then exposed SC to 3 log_10_ focus-forming units of ZIKV strain H/PF/2013, which is a human isolate from the 2013 French Polynesia outbreak [39] or strain Paraíba 2015, an isolate from Brazil [55]. ZIKV replication was observed in several organs of mice treated with the antibody; however, the mice did not lose weight, succumb to infection, or develop neuropathology. The same group established an in utero transmission model of ZIKV infection using wild-type mice treated with this IFNAR-1 blocking monoclonal antibody [56]. In another report, IFN blockade by the same monoclonal antibody followed by SC infection with a higher dose of an African lineage strain of ZIKV (Dakar 41519) that was derived from a brain homogenate via passage in *Rag1*^-/-^ mice resulted in lethal disease [43]. Our mouse model results are similar to those in this report in that we found that antibody blockade of the type I IFN receptor recapitulates the severity of ZIKV disease observed in *Ifnar1*^-/-^ mice. Further, we demonstrated that route of exposure is a factor in lethality in this mouse model in that mice exposed by the IP route began to succumb on day 7 PI and 100% mortality was observed by day 12 PI. In contrast, 40% mortality was observed when mice were exposed SC and they succumbed as late as day 19 PI. However, the disease was similar in mice that succumbed to ZIKV regardless of the route of exposure where significant pathologic changes were observed in the CNS. Since vaccination studies requiring boosting would utilize older mice, we confirmed that lethality is observed in mice that are 10 weeks old where 80–100% mortality was observed in mice exposed to 6 log_10_ PFU IP of ZIKV strain DAK AR D 41525 used in this study. These results are being submitted in a separate publication that also evaluates the susceptibility of this mouse model to multiple ZIKV strains. Zhao et al. report higher lethality by SC infection which may be due in part to passaging their virus in mice [43]. Our results and those described by Zhao et al. and Lazear et al. suggest that the virus strain (African vs. Asian lineage), passage history, and exposure route can affect susceptibility to infection. More studies are needed to evaluate the pathogenesis of African vs. Asian lineage strains (and the effect of passage history) in the various infection models.

Mortality is not a common feature of human infection with ZIKV, which is generally asymptomatic and self-limiting in most individuals. However, severe disease in humans is characterized by neurological complications associated with ZIKV infection. In adults, reported neurological complications include GBS [24,25] or in a few cases, encephalopathy [57], meningoencephalitis [58], and acute myelitis [59] have been described. In the fetus, intrauterine infection can cause congenital abnormalities to include severe fetal brain injury [22]. The neuropathology that we observed in our studies offers a model for dissecting the pathological consequences of ZIKV infection. We observed significant pathologic changes in the CNS of all mice that succumbed to ZIKV infection. Overall, the CNS lesions in these mice represent an acute to subacute encephalitis/encephalomyelitis that is characterized by neuronal death, astrogliosis, microgliosis, scattered necrotic cellular debris, and a minimal to mild mononuclear (and less frequently neutrophilic) inflammatory cell infiltrate. In the brain, lesions were most evident in the hippocampus, particularly affecting the pyramidal and granule cell layers, followed by thalamus and cerebrum, and less often affecting the cerebellum and pons. The presence of ZIKV RNA as detected via ISH suggests these lesions are attributable to ZIKV infection. It is likely that encephalitis/encephalomyelitis contributed to the morbidity and mortality in these animals, particularly those that succumbed early, between days 7 to 11 PI. Lesions, particularly neuronal necrosis and inflammation, appear to be less severe in animals from day 12; however, it is possible that CNS injury played a role in the deaths of these animals as well. The neurotropism of ZIKV was demonstrated in early studies where neonatal mice intracerebrally infected with ZIKV showed evidence of nuclear fragmentation, perivascular cuffing, and degenerative cells in the hippocampus of the brain [3,28]. Bell et al. also observed enlarged astrocytes with extended processes and containing cytoplasmic virus factories throughout the cortex of ZIKV-infected mouse brains [28]. More recent studies characterizing ZIKV murine models in immunodeficient mice also showed that microscopic lesions resulting from ZIKV infection were found primarily in the brain [37,38,40]. Our findings are most consistent with those reported by Dowall et al. [38] where inflammatory and degenerative changes were observed in the brains of A129 mice challenged with ZIKV. The CNS lesions observed in ZIKV-infected mice may be relevant for brain-related pathologies in some ZIKV-infected humans. However, the histopathology of ZIKV-associated microcephaly has been limited to only a few reports thus far (reviewed in [60]). The major findings have mostly been in the brain and include diffuse grey and white matter involvement consisting of dystrophic calcifications, gliosis, microglial nodules, neuronophagia, and scattered lymphocytes [60]. Astrocyte pathology was observed in post-mortem analysis of a neonatal brain with microcephaly associated with ZIKV infection where diffuse astrogliosis was apparent with focal astrocytic outburst into the subarachnoid space. Activated microglial cells were also found to be present throughout most of the cerebral gray and white matter [22].

Outside of the CNS, the most consistent histopathological lesions were observed in the spleen. We observed lymphocytolysis in the splenic white pulp which correspond to areas with ZIKV ISH signal. Dowall et al. also noted similar lesions in the spleen although detection of viral RNA was not described [38]. Other histopathological observations indicated a significant inflammatory response resulting from ZIKV infection. Lymphoid hyperplasia and myeloid (granulocytic) hyperplasia were noted and are indicative of an inflammatory response to antigenic stimulation and an increased demand for leukocytes. Although these are both somewhat non-specific findings, in these cases they most likely represent a systemic immune reaction to ZIKV infection and are related to the significant neuroinflammation noted in the animals. A previous study indicated that ZIKV infection in AG129 mice led to a systemic inflammatory response, where multiple pro-inflammatory cytokines were found to be increased in the sera [40]. It is unknown how this relates to human disease since the cytokine response during the acute phase of ZIKV infection in humans has not been studied.

Inflammation was also observed in the skeletal muscles of the head and vertebral column. Skeletal muscle degeneration and inflammation observed in these animals are thought to be attributable to ZIKV infection since direct infection of the myocytes was observed on day 3 PI. ZIKV is presumably cleared following day 3 PI, but inflammation persists in the skeletal muscle. Interestingly, Ailota et al. described similar pathologic changes in the musculature from the posterior rear limb of a ZIKV-infected mouse where multi-focal myofiber degeneration and necrosis with inflammatory cell infiltration, nuclear rowing, and attempted regeneration were observed. Further investigation into whether the skeletal muscle inflammation is viral or immune mediated is warranted.

In summary, we described a murine model for ZIKV that mimics the severe neurological disease previously described in mice deficient in the type I or II IFN response [37,38,40]. Our detailed description of the ZIKV-associated pathology in this model, much of which mirrors what is known about neuropathogenesis in humans, will provide a baseline for evaluating medical countermeasures to prevent or treat ZIKV infections.

## Supporting Information

S1 Table Significant Microscopic Findings in ZIKV-Infected Mice Treated with an IFNAR1-Blocking MAb. FD indicates mice that were found dead and E indicates mice that were euthanized. The semi-quantitative scale is indicated by the following scores: 0 = none; 1 = minimal; 2 = mild; 3 = moderate; 4 = marked; 5 = severe.
(DOCX)

S1 Fig ISH and IFA Findings in the Kidney of ZIKV-Infected Wild-type Mice Treated with an IFNAR1-Blocking MAb or Uninfected Control Mice. (A) Representative ISH staining demonstrating that ZIKV RNA is detected in the muscle cells of a blood vessel in the kidney of a mouse exposed to ZIKV IP that succumbed on day 7 PI; scale bar represents 50 μm. (B) Representative ISH staining demonstrating no ZIKV RNA is detected in the kidney of an uninfected control mouse; scale bar represents 100 μm. (C) IFA confirmed the presence of ZIKV in the smooth muscle (SMA) of a blood vessel in the kidney of a mouse exposed to ZIKV IP that succumbed on day 7 PI. (D) IFA did not detect ZIKV in the kidney from uninfected control mice; scale bar represents 20 μm. (E) Isotype control antibody staining in the kidney of a mouse exposed to ZIKV IP that succumbed on day 7 PI; scale bar represents 20 μm. The findings in the kidney are from one independent experiment where a total of 11 ZIKV-infected mice (3 uninfected controls) were analyzed. All sections were analyzed by an unblinded, board-certified veterinary pathologist.
(TIF)

S2 Fig Histologic and ISH Findings in the Skeletal Muscle of ZIKV-Infected Wild-type Mice Treated with an IFNAR1-Blocking MAb or Uninfected Control Mice. (A) Hematoxylin and eosin staining showed myocyte degeneration, inflammation, and nuclear rowing (indicated by the arrows) in the vertebral column skeletal muscle of a mouse exposed to ZIKV IP that was euthanized on day 12 PI; scale bar represents 100 μm. (B) Hematoxylin and eosin staining showed multifocal myocyte degeneration and inflammation (indicated by asterisks) in the skeletal muscle of the head of a mouse exposed to ZIKV IP that succumbed on day 11 PI; scale bar represents 50 μm. (C) ISH staining demonstrating that ZIKV RNA is detected in the skeletal muscle cells of a mouse exposed to ZIKV IP that was euthanized on day 3 PI; scale bar represents 200 μm. (D) Representative ISH staining demonstrating no ZIKV RNA is detected in the skeletal muscle of an uninfected control mouse; scale bar represents 200 μm. (E) Representative hematoxylin and eosin staining in the skeletal muscle of an uninfected control mouse; scale bar represents 100 μm. The findings in the skeletal muscle are from two independent experiments where a total of 16 ZIKV-infected mice (3 uninfected controls) were analyzed. All sections were analyzed by an unblinded, board-certified veterinary pathologist.
(TIF)
