# Supplementary figures and images for: Neuropathogenesis of Zika Virus in a Highly Susceptible Immunocompetent Mouse Model after Antibody Blockade of Type I Interferon
Source: PLoS Negl Trop Dis. 2017 Jan 9;11(1):e0005296. doi: 10.1371/journal.pntd.0005296 (PMC5249252; doi:10.1371/journal.pntd.0005296)

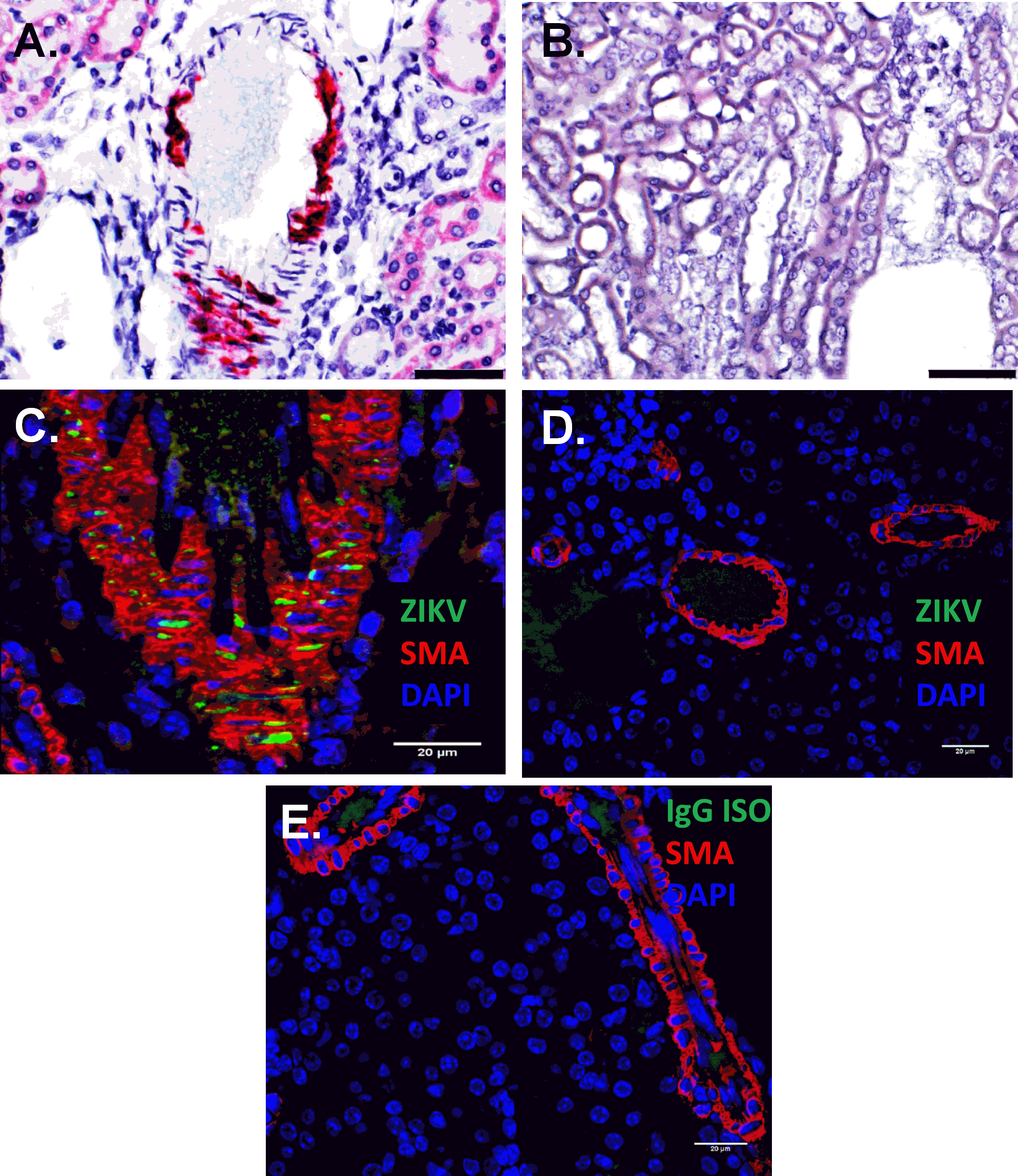

Supplement: S1 Fig — (A) Representative ISH staining demonstrating that ZIKV RNA is detected in the muscle cells of a blood vessel in the kidney of a mouse exposed to ZIKV IP that succumbed on day 7 PI; scale bar represents 50 μm. (B) Representative ISH staining demonstrating no ZIKV RNA is detected in the kidney of an uninfected control mouse; scale bar represents 100 μm. (C) IFA confirmed the presence of ZIKV in the smooth muscle (SMA) of a blood vessel in the kidney of a mouse exposed to ZIKV IP that succumbed on day 7 PI. (D) IFA did not detect ZIKV in the kidney from uninfected control mice; scale bar represents 20 μm. (E) Isotype control antibody staining in the kidney of a mouse exposed to ZIKV IP that succumbed on day 7 PI; scale bar represents 20 μm. The findings in the kidney are from one independent experiment where a total of 11 ZIKV-infected mice (3 uninfected controls) were analyzed. All sections were analyzed by an unblinded, board-certified veterinary pathologist. (TIF) [file pntd.0005296.s002.tif]

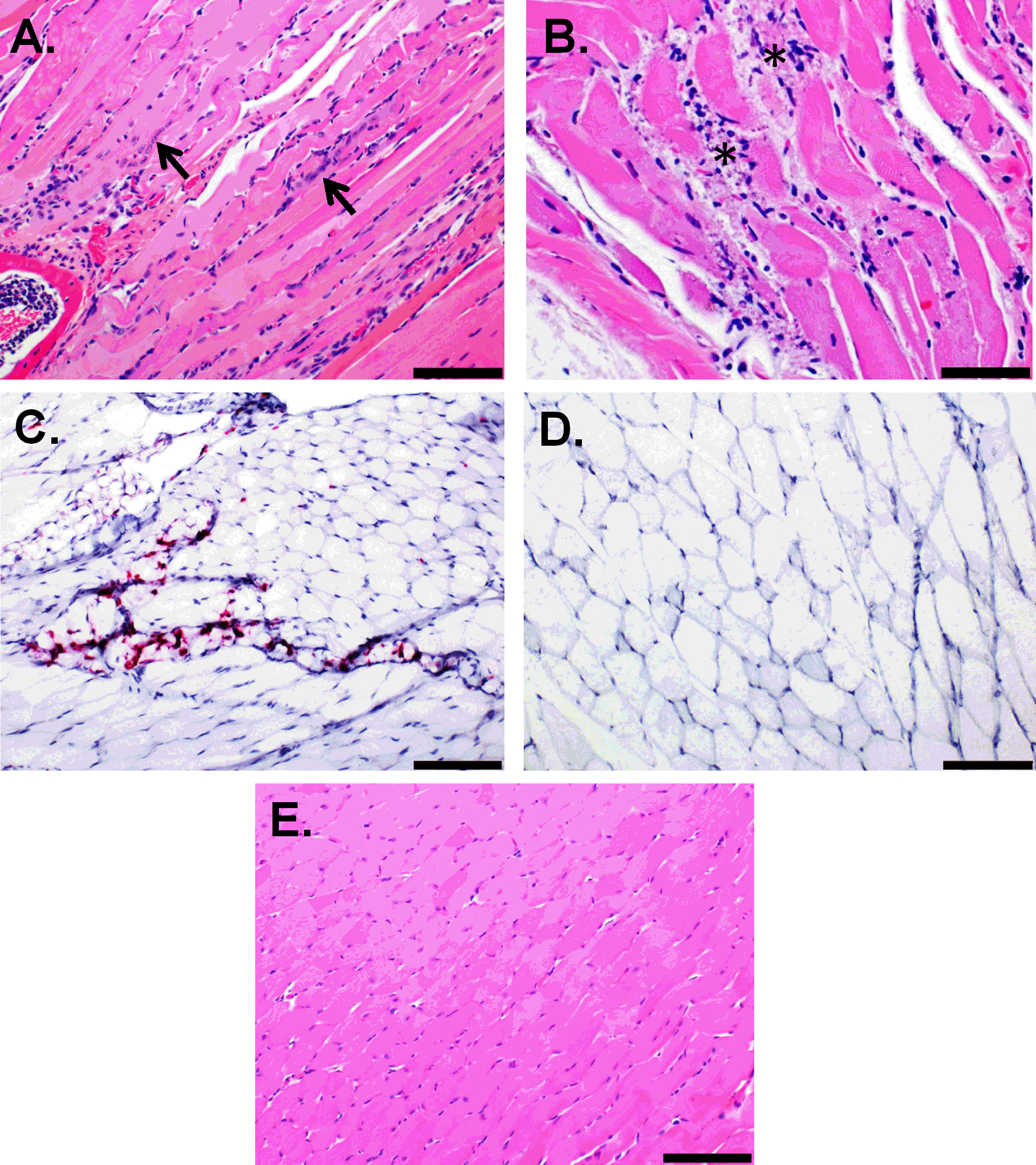

Supplement: S2 Fig — (A) Hematoxylin and eosin staining showed myocyte degeneration, inflammation, and nuclear rowing (indicated by the arrows) in the vertebral column skeletal muscle of a mouse exposed to ZIKV IP that was euthanized on day 12 PI; scale bar represents 100 μm. (B) Hematoxylin and eosin staining showed multifocal myocyte degeneration and inflammation (indicated by asterisks) in the skeletal muscle of the head of a mouse exposed to ZIKV IP that succumbed on day 11 PI; scale bar represents 50 μm. (C) ISH staining demonstrating that ZIKV RNA is detected in the skeletal muscle cells of a mouse exposed to ZIKV IP that was euthanized on day 3 PI; scale bar represents 200 μm. (D) Representative ISH staining demonstrating no ZIKV RNA is detected in the skeletal muscle of an uninfected control mouse; scale bar represents 200 μm. (E) Representative hematoxylin and eosin staining in the skeletal muscle of an uninfected control mouse; scale bar represents 100 μm. The findings in the skeletal muscle are from two independent experiments where a total of 16 ZIKV-infected mice (3 uninfected controls) were analyzed. All sections were analyzed by an unblinded, board-certified veterinary pathologist. (TIF) [file pntd.0005296.s003.tif]
