# Supplementary material for: Neuropathogenesis of Zika Virus in a Highly Susceptible Immunocompetent Mouse Model after Antibody Blockade of Type I Interferon
Source: PLoS Negl Trop Dis. 2017 Jan 9;11(1):e0005296. doi: 10.1371/journal.pntd.0005296 (PMC5249252; doi:10.1371/journal.pntd.0005296)
Supplement: Supplementary file 2 [file pntd.0005296.s001.docx]

	Animal #	M1/IP	M2/ IP	M3/ IP	M4/ IP	M1/ SC	M5/ IP	M6/ IP	M7/ IP	M8/ IP	M9/ IP	M10/ IP
	Day PI	7	7	7	8	8	11	11	11	11	12	12
	Comments	FD	E	E	E	E	FD	E	E	E	E	E
Organ	Lesion											
Brain	Perivascular cuffing	2	1	2	1	2	0	0	1	0	0	1
Brain	Microgliosis	2	2	2	2	2	1	1	2	1	1	1
Brain	Neuronal necrosis	1	2	1	3	0	0	1	3	1	0	0
Brain	Scattered necrotic debris	1	1	2	2	2	0	1	1	1	1	1
Brain	Neutrophilic infiltrates	1	0	0	1	0	0	1	0	1	1	0
Brain	Neuropil vacuolation (edema)	2	1	1	1	1	0	1	1	0	0	0
Spinal cord	Perivascular cuffing	1	1	1	n/a	2	n/a	n/a	0	0	0	0
Spinal cord	Microgliosis	3	2	1	n/a	2	n/a	n/a	1	1	0	1
Spinal cord	Satellitosis	0	1	0	n/a	1	n/a	n/a	0	1	0	0
Spinal cord	Neuronal necrosis	1	1	1	n/a	1	n/a	n/a	1	1	0	0
Spinal cord	Neuropil Vacuolation (edema)	2	1	1	n/a	2	n/a	n/a	1	1	0	0
Spinal ganglion	Necrosis	2	0	0	n/a	0	n/a	n/a	0	0	0	0
Spleen	Lymphocytolysis	2	2	2	1	1	n/a	n/a	1	1	n/a	1
Spleen	Lymphoid hyperplasia	1	1	0	0	1	n/a	n/a	1	0	n/a	0
Skeletal muscle, head	Inflammation	0	1	0	2	1	1	1	1	1	0	0
Skeletal muscle, head	Degeneration	0	1	0	1	1	2	1	1	1	0	0
Skeletal muscle, head	Regeneration	0	0	0	1	0	0	1	1	1	0	0
Skeletal muscle, vertebral	Inflammation	2	1	1	n/a	2	2	3	1	2	1	1
Skeletal muscle, vertebral	Degeneration	1	1	1	n/a	1	2	1	1	1	1	1
Skeletal muscle, vertebral	Regeneration	0	1	0	n/a	0	0	1	0	1	1	1
Score:	0= none 1=minimal, 2=mild, 3=moderate, 4=marked, 5=severe											
	Artefactual damage precludes adequate assessment of tissue											
	SC route of challenge											
